# Competing Risks of Coronary Heart Disease Mortality versus Other Causes of Death in 10 Cohorts of Middle-Aged Men of the Seven Countries Study Followed for 60 Years to Extinction

**DOI:** 10.3390/jcdd10120482

**Published:** 2023-11-30

**Authors:** Paolo Emilio Puddu, Paolo Piras, Anthony Kafatos, Hisashi Adachi, Hanna Tolonen, Alessandro Menotti

**Affiliations:** 1 Association for Cardiac Research, 00182 Rome, Italy; amenotti2@gmail.com; 2 EA 4650, Signalisation, Électrophysiologie et Imagerie des Lésions d’Ischémie Reperfusion Myocardique, Université de Normandie, 14032 Caen, France; 3 Department of Structural Engineering, Sapienza University of Rome, 00185 Rome, Italy; paolo.piras@uniroma3.it; 4 Department of Social Medicine, Preventive Medicine and Nutrition Clinic, University of Crete, 70013 Heraklion, Greece; kafatos@med.uoc.gr; 5 Department of Internal Medicine, Division of Cardio-Vascular Medicine, School of Medicine, Kurume University, Kurume 830-0011, Japan; hadac@med.kurume-u.ac.jp; 6 Department of Public Health and Welfare, Finnish Institute for Health and Welfare, FI-00271 Helsinki, Finland; hanna.tolonen@thl.fi

**Keywords:** competing risks, Fine-Gray, Cox, mortality, CHD, stroke, Heart Diseases of Uncertain Etiology, CVD, risk factors, male cohorts, 60-year follow-up

## Abstract

Objectives: To assess whether competing risks help explain why regions with initially high serum cholesterol have higher mortality from coronary heart disease (CHD) and lower mortality from stroke and other major heart diseases, while the reverse is found for those with initially lower serum cholesterol. Material and Methods. Ten cohorts of men (N = 9063) initially aged 40–59 in six countries were examined and followed for fatal outcomes for 60 years. Major cardiovascular disease (CVD) groups were CHD, stroke, and other Heart Diseases of Uncertain Etiology (HDUE), or the combination of stroke and HDUE (STHD), along with all other causes of death. Fine-Gray competing risk analysis was applied with CHD versus all other causes of death or STHD (direct mode) and all other causes of death or STHD versus CHD (inverse mode), and the effects of 19 covariates (of which 3 references) on the cause-specific hazard of the outcomes were assessed, thus investigating potential etiologic roles. A systematic comparison with results obtained by running the Cox model in direct and inverse modes with the same end-point results was also performed and illustrated graphically. Results. CHD mortality is bound to different risk factor relationships when compared with all other causes of death and with STHD. The role of serum cholesterol is crucial since, in both comparisons, by Fine-Gray, its coefficients are positive and significant for CHD and negative and significant for all other causes of death and STHD. Risk factor capabilities in specific outcome types of the CVD domain (CHD versus STHD) are different depending on the outcome types considered. Risk factor coefficients are smaller in Fine-Gray modelling and larger in the Cox model. Fine-Gray detects different risk factors whose coefficients may have opposite algebraic signs. Conclusions. This is the first report whereby a large group of risk factors are investigated in connection with life-long CVD outcomes by Fine-Gray competing risk analysis, and a systematic comparison is performed with results obtained by Cox models in both direct and inverse modes. Subtypes of CVD mortality should be summed with full awareness that some risk factors vary by pathology, and they should at least be disentangled into CHD and STHD.

## 1. Introduction

The Seven Countries Study of Cardiovascular Diseases (SCS) started in the late 1950s–early 1960s, and 10 of the 16 cohorts of middle-aged men initially enrolled have been followed for 60 years to their practical extinction [1,2,3]. In the most recent analysis [4], we showed that SCS cohorts with initially high average serum cholesterol levels had higher mortality from coronary heart disease (CHD) and lower mortality from stroke and other major heart diseases, while the reverse was true for cohorts with initially lower serum cholesterol, where CHD mortality was low while other deaths were higher.

We posed the question of whether a competing risk model whereby CHD outcome versus other fatal events were compared [4] might help to better understand the patterns of mortality in cohorts followed by extinction. Our analyses incorporate information about competing risks using Fine-Gray methods introduced in 1999 [5,6] and previously applied in part of the SCS with shorter follow-up [7,8]. Although competing risk techniques were a topic for recent editorials or reviews [9,10,11,12] rather than original investigations [13,14,15,16], we believe it is important to apply them in cohorts followed for life to examine how they compare with standard modeling strategies such as applying the Cox model. This is conducted here, for the first time, in the SCS, a prototype of epidemiological research. We hypothesized that there are important differences in pathology among CHD, stroke, and congestive heart failure (CHF), such that total cardiovascular disease (CVD) death as a combined outcome should be studied with full awareness of these differences [4].

## 2. Materials and Methods

**Populations and measurements.** The analysis was run on 10 of the 16 cohorts of the SCS that were followed for mortality for 60 years. The cohorts were the US Railroad workers; two rural cohorts in Finland (East and West Finland); one sample of men from the town of Zutphen—the Netherlands; two rural cohorts in Italy (Crevalcore and Montegiorgio); two rural cohorts in Greece (Crete and Corfu); one cohort from a rural village and one from a fishing village in Japan (Tanushimaru and Ushibuka). Men were aged 40 to 59 years between 1958 and 1961 when this investigation started, and the participation rate was very high (around 95%). More details are provided elsewhere [1,2,3].

Risk factors measured and used in this analysis were: (a) age, in years approximated to the nearest birthday; (b) physical activity at work, derived from questions combined and related to the reported occupation, classified as sedentary, moderate, or vigorous (in %); in one country, this classification was validated by ergonometric measurements [17] and energy intake derived from dietary history [18]; (c) smoking habits were derived from a questionnaire and classified as never smokers, ex-smokers, and current smokers (in %); (d) body mass index, derived from height and weight measured according to the procedure of the WHO Cardiovascular Survey Methods Manual (WHO Manual), in kg/m^2^ [19]; (e) systolic blood pressure measured in supine position at the end of a physical examination using mercury sphygmomanometers and following the procedure proposed in the WHO Manual [19] (in mmHg); the average of two measurements taken one minute apart was used for analysis; (f) heart rate, derived from a resting ECG (in beats/min); (g) serum cholesterol measured on casual blood samples following the technique of Anderson and Keys [20]; (h) prevalence of cardiovascular diseases (CVD) according to the criteria used to define CVD prevalence in the SCS [1] expressed as 1 = yes; 0 = no; (i) prevalence of silent major ECG abnormalities in subjects without a diagnosis of CVD, defined as positive in the presence of any of the following codes of the Minnesota Code, edition 1968 [19]: 1.1, 1.2, 5.1, 5.2, 6.1, 7.1, 7.4, 8.3, corresponding to major Q waves, major negative T waves, 3rd degree AV block, left ventricular branch or intraventricular block, atrial fibrillation; expressed as 1 = yes, 0 = no.

Life status and mortality were checked during 60 years, and, among 9063 men, there were censored cases (0.8%) only for 3 subjects who were still alive and 68 lost to follow-up. Therefore, the analysis was conducted on 99.2% of men enrolled at baseline (N = 8992).

Causes of death were allocated by reviewing death certificates and frequently combining that information with data from interim examinations, hospital and medical records, interviews with physicians and relatives of the deceased, and other witnesses to fatal events. Causes of death were assigned by a single coder (AM) following defined criteria and using the 8th revision of the WHO-ICD (ICD-8) [21]. In cases of multiple causes and uncertainty about the principal cause, a hierarchical preference was adopted, with violence, cancer, CHD, stroke, and other causes in that order.

Cardiovascular mortality end-points were chosen as follows: (1) coronary heart disease (CHD), including cases of myocardial infarction, acute ischemic heart attacks, and sudden coronary death, after the exclusion of other possible causes (ICD-8 codes 410, 411, 412, 413, 795); cases with only mention or evidence of chronic coronary heart diseases (part of code 412) were not included in this group for reasons given elsewhere [22] while healed myocardial infarction was retained in this group; (2) Cerebrovascular diseases (stroke) included any type of cerebrovascular disease (ICD-8 codes 430–438); (3) Heart disease of uncertain etiology (HDUE) included a pool of symptomatic heart diseases (ICD-8 code 427 corresponding to heart failure, arrhythmia, blocks), ill-defined hypertensive heart disease (usually in the absence of documented left ventricular hypertrophy) (ICD-8 codes 402–404), and cases classified as chronic or other types of coronary heart disease, without the presence of typical coronary syndromes (ICD-8 parts of codes 412 and 414); usually manifested with heart failure, arrhythmia, and blocks. The pool of these three selected CVD end-points covered 92% of all CVD deaths in 60 years. Rare or etiologically defined cardiovascular diseases were not included in this analysis. We also combined stroke rates with HDUE rates to obtain a single counterpart (STHD) versus CHD.

Baseline measurements were taken before the era of the Helsinki Declaration, and approval was implied in participation, while verbal or written consent was obtained for the collection of follow-up data.

**Statistical analysis.** Analyses were run on data after combining cohorts in the same country. About 4 per 1000 baseline risk factor measurements were missing and were imputed by the multivariate normal procedure. Baseline risk factor measurements for the 6 countries were described as means or proportions for each country and on total cases and compared across cohorts by ANOVA or chi-squared test.

The death rates for the 3 CVD end-points, the combined end-point STHD, and the pool of all 3 CVD end-points were computed for the 60 years of follow-up in all cohorts combined and separately in the 6 regions. The same tabulation includes rates for all-cause mortality in each of the six regions.

To study the risks in competition, we used the Fine-Gray technique, which represents a variation of the Cox model for proportional hazards, including the sub-distribution of a competing risk [5], using the R package as described by Gray [6].

Competitive events may be studied in pairs. We used two approaches. *Approach 1*: The events were CHD deaths (CHD) as the primary event and all other causes of death (OTHERS) as competing events, with the exclusion of the 3 survivors and the 68 lost to follow-up. Two models were produced: (a1) the direct model with CHD as the primary event and OTHERS as the competing event, and (b1) the inverse model with OTHERS as the primary event and CHD as the competing event; *Approach 2*: The events in competition were CHD and the sum of stroke plus HDUE deaths (STHD), with the exclusion of all other participants. The two models were: (a2) the direct model with CHD as a primary event and STHD as a competing event; and (b2) the inverse model with STHD as a primary event and CHD as a competing event.

In these cohorts, followed by extinction, there are many competing events. This may increase the difference between the Fine-Gray model and the more classical Cox model. A systematic comparison between the Fine-Gray and Cox models was also performed. The Cox models were run using the identical approaches used for the Fine-Gray models run above and with identical eliminations and inversions. A synoptic graphical figure illustrated the most striking differences between Cox and Fine-Gray methods when CHD versus OTHERS and CHD versus STHD were compared along the respective inversion of the primary event roles.

The same risk factors shown in Table 1 were used as covariates, and coefficients and hazard ratios (with 95% confidence intervals) were calculated, while statistical significance was ascertained at *p* ≤ 0.05. In comparing Fine-Gray and Cox results, only coefficients and standard errors (SE) along with *t*-tests were used, which were also considered for the synoptic graphical illustration.

## 3. Results

The mean levels of risk factors at entry examination for the 6 countries are summarized in Table 1, where a large heterogeneity across countries was documented and tested by ANOVA and Chi-squared tests. See also Table A1 in Appendix A for continuous risk factors expressed as medians and interquartile percentiles (25–75). Most factors have, expectedly, higher levels in the USA and Northern European countries and lower levels in Southern Europe and Japan. However, some exceptions are evident, such as the low prevalence of sedentary physical activity in Finland and the high prevalence of smokers in Japan. The largest difference was observed for mean serum cholesterol, with a maximum difference across countries of almost 100 mg/dL. More details can be found in previous contributions [1,2,3].

Major CVD-type 60-year death rates are described in Table 2 for all 6 countries and their pool among 9063 middle-aged men. A large heterogeneity of individual causes of death rates is evident for the major causes considered: CHD, stroke, and HDUE. This is well illustrated by the ratio of CHD/STHD in Northern European countries and the USA, where CHD death rates were the highest while stroke and HDUE death rates were the lowest. The reverse pattern was seen in Southern European countries. Japan was singular, with the highest death rates for stroke and the lowest for CHD and HDUE. On the other hand, the pool showed that considering all 3 major CVD types (CVD), there were 4 countries with rates under the averaged value of 441 per 1000 in 60 years (the Netherlands, Italy, Greece, and Japan), whereas 2 countries (the USA and Finland) had values far outside that threshold.

Table 3 and Table 4 illustrate, respectively, the complete model of CHD versus all other causes of death and the model of CHD versus STHD (other causes excluded), thus named partial, both adjusted for competing risks:

*Approach 1:* The model with CHD as the principal event (complete direct model: Table 3 (A)) had 5 risk factors with significant positive coefficients (smoker, BMI, systolic blood pressure, serum cholesterol, and prevalent CVD). Moreover, dummy variables identifying countries had significant negative coefficients in the USA, the Netherlands, Italy, Greece, and Japan (lower CHD death rates versus Finland as a reference). The model with all other causes of death as the principal event (complete inverse model: Table 3 (B)) had 7 risk factors with significant coefficients (age, ex-smokers, smokers, and heart rate [positive] and BMI, systolic blood pressure, and serum cholesterol [negative]). Dummy variables for countries had significant, positive coefficients in Italy, Greece, and Japan.

Comparing all the coefficients of these 2 models showed that 5 were significantly different (age, smoker, systolic blood pressure, serum cholesterol, and CVD prevalence), plus 3 of those related to the country dummy variables (those negative in the first model becoming positive in the second model).

In general, in the complete inverse model, the coefficient of age was significantly larger, that of smokers was smaller, and that of heart rate had an opposite algebraic sign [positive], and those of systolic blood pressure, serum cholesterol, and prevalent CVD were smaller. Most importantly, the coefficients of serum cholesterol and prevalent CVD had negative algebraic signs in opposition to the model with CHD as the principal event, where they were expectedly positive.

*Approach 2:* The model, with CHD as the principal event (partial direct model: Table 4 (A)), had 4 risk factors with significant coefficients (age [negative], smoker, serum cholesterol, and prevalent CVD [positive]). Moreover, dummy variables identifying countries had significant negative coefficients in the USA, Italy, Greece, and Japan (lower CHD death rate versus Finland). The model with STHD as the principal event (partial inverse model: Table 4 (B)) had 5 risk factors with significant coefficients (age [positive] and smoker, serum cholesterol, ECG abnormalities, prevalent CVD [all negative]) and positive significant coefficients for dummy variables for the USA, Italy, Greece, and Japan.

Comparing the coefficients of these 2 models indicated that 4 were significantly different (age, smoker, serum cholesterol, CVD prevalence), plus 3 of those related to the country dummy variables.

In general, in the partial inverse model of STHD, the coefficients of age, smoker, serum cholesterol, and CVD prevalence were significantly larger. However, the most interesting aspect was that, again, like in Table 3 of the complete models, the algebraic signs of the coefficients were opposite in the inverse versus the direct partial models (positive for age and negative for smoker, serum cholesterol, and CVD prevalence). In addition, the coefficients of dummy variables for countries became positive instead of negative in the USA, Italy, Greece, and Japan, with a largely significant difference.

The coefficients and algebraic signs of Table 3 and Table 4 were compared with those of the direct and partial Cox models in Table 5. When the 4 columns of *t*-tests are considered in the comparison between Cox and Fine-Gray coefficients, there are statistically significant differences for age, smoker, systolic blood pressure, serum cholesterol, and CVD prevalence for the majority of outcomes, whereas only a few were different for moderate physical activity, heart rate, and the dummy variables for countries. Cox coefficients were constantly larger than Fine-Gray ones. However, there were statistically significant coefficients by Fine-Gray that were not by Cox.

**Figure 1:**
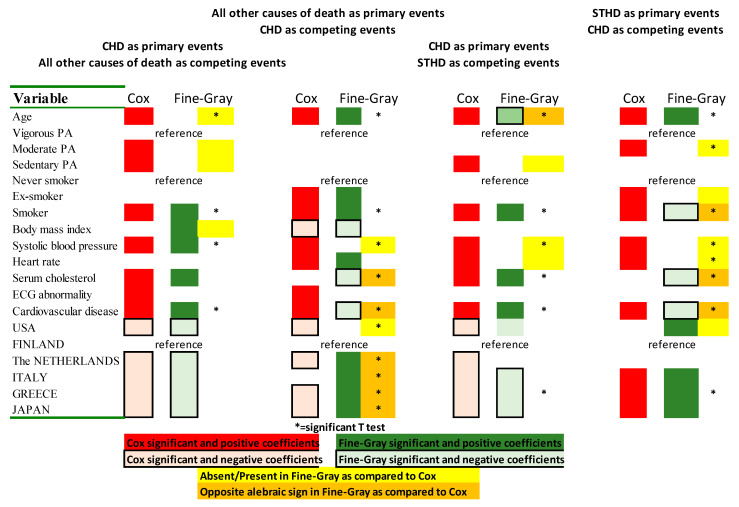
Synoptic graphical illustration of the differences between Cox and Fine-Gray models.

Figure 1 illustrates synoptically the main differences between significant positive Cox coefficients (dark red) versus significant positive Fine-Gray coefficients (dark green) along the respective negative coefficients (light red and light green), pointing to the statistically significant differences (asterisks), the absence/presence in one model versus the other (yellow), or the opposition in the algebraic sign of the coefficients (orange). The most striking differences are: (1) the opposite algebraic signs for serum cholesterol and CVD prevalence in OTHERS versus CHD and STHD versus CHD, all statistically significant; (2) the opposite algebraic signs for age and smoker, respectively in CHD versus STHD and STHD versus CHD; (3) absence/presence between Cox and Fine-Gray models of: (a) age (statistically significant), moderate and sedentary physical activity and body mass index in CHD versus OTHERS; (b) systolic blood pressure (statistically significant) in OTHERS versus CHD; (c) sedentary physical activity, systolic blood pressure (statistically significant) and heart rate in CHD versus STHD; (d) moderate physical activity (statistically significant), ex-smoker and systolic blood pressure and heart rate (both statistically significant) in STHD versus CHD; (4) absence/presence differences or opposition in the algebraic signs for dummy variables defining countries in part of these comparisons.

## 4. Discussion

This is the first report whereby several risk factors are investigated in connection with life-long CVD outcomes by Fine-Gray methods for competing risk analysis. Findings summarized in Table 3 and Table 4 suggest that CHD mortality is bound to different risk factor relationships when compared with all other causes of death and with STHD. The most evident difference remains in the role of serum cholesterol since in both comparisons its coefficients are positive and significant for CHD and negative and significant for the other end-points (all other causes of death and STHD), thus presumably indicating that etiologies are different, which reinforces previously submitted hypotheses [4,7,8]. This is the largest residential cohort of men enrolled in different geographic areas followed-up life-long for 3 types of CVD death and indicates the need to take competing risks into account to investigate individual outcomes.

Another aspect is provided by the coefficients of the dummy variables identifying the countries in SCS, since in the low-cholesterol areas they are negative and significant for CHD while they become positive and significant for the other end-points. Again, this evidence points out that CVD death rates, largely different among countries (Table 2), should not be equated [4]. The different risk factors implicate (Table 3 and Table 4) that there are also different etiologies. Specific interventions can only have an effect on the end-point they address, and research questions might focus on risk factors for a certain outcome [9]. However, all relevant pathways should be considered in efforts to extend life.

All differences shown in Table 5 indicate that Cox coefficients are larger than Fine-Gray ones, with the exception of the dummy variables defining Greece, where they are smaller. Moreover, in the inverse model comparison of Table 3, all country dummy variables had smaller coefficients by Fine-Gray. These systematic statistical differences corroborate and complement those observed in Table 3 and Table 4 on the specific importance of selecting which one is the primary event type considered and thus the need to take competition of risks into proper account, which is not the case by Cox. Figure 1 extends and reinforces the striking differences observed by comparing the Cox and Fine-Gray models side by side and points to the need to consider several covariates concomitantly and the competing risk analysis to correctly define outcomes in the CVD death area.

There are two different families of models when regression models are fitted in the presence of competing risks (by Fine-Gray) [11,12]: (a) modeling the covariates on the cause-specific hazard of the outcome or (b) modeling the covariates on the cumulative incidence function. Whereas the (a) models how the covariates might impact the rate of occurrence of the outcome in those subjects who are currently event-free, the (b) models estimate the effect of covariates on the absolute risk of the outcome over time. Etiologic questions may well be addressed by the former family of models, as we did previously [8,9] and present in this investigation, whereas patients’ clinical prognosis may be estimated better using the latter family of models [10].

Risk analysis. The literature offers important contributions from the 1999 initial description by Fine and Gray of the basic methodologies to obtain competing risk analyses [6,7]. However, much is made by editorialists and reviewers to concentrate on the use and interpretation of the Fine-Gray sub-distribution hazard model and the fact that in survival analysis, a competing risk is an event whose occurrence precludes the occurrence of the primary event of interest [9,10,11,12]. There have been, on the other hand, very few contributions dealing with cohorts followed up long enough to reach extinction and/or analyzing a large series of risk factors for a proper comparison with our findings [13,14,15,16].

On the other hand, there is no literature where a systematic comparison was performed between standard Cox and Fine-Gray models using the reverse mode of analysis, as we did here, whereby the former primary end point was reversed to become a secondary one. The idea came from the correlation concepts whereby one may invert Y (dependent variable) and X (independent variable), and the purpose was to moderate the immortality bias inherent to the competing risk analysis by Fine-Gray, where one subject experiencing the primary event cannot also experience the secondary one. There are indeed large differences among predictive covariates (Table 3, Table 4 and Table 5), not only in terms of the presence/absence and opposition of the coefficients’ algebraic signs when primary end-points become secondary end-points, and this is more evident by Fine-Gray model applications than by Cox (see Figure 1). Whether the cause of these striking results relies on the immortality limits of Fine-Gray or the absence of care for competitions by Cox is still an obscure question. Clearly, much more comparative investigations are needed with multi-covariate studies and quite long follow-up with both sexes to accrue a sufficiently large number of events.

A brief review of the 2012-23 literature [13,14,15,16] enables us to conclude that there were relatively few investigations, generally studying a short series of covariates (one or two at maximum), short follow-ups, and only primary versus secondary outcome analyses of competition, with the exception of our group concentrating on one cohort of the SCS, quite a long follow-up with several covariates considered, and both direct and inverse modes of analysis to assess competition and its impact on outcomes [7,8].

Feinstein et al. [13] used in 2012 the competing risks model described by Fine and Gray to assess the association of race with the cumulative incidence of five competing outcomes: CHD (defined as non-fatal myocardial infarction or CHD death), fatal/non-fatal stroke, heart failure, other CVD deaths, and other non-CVD causes of death within and between races in three multi-center, NHLBI-sponsored cohorts. Among 45–64-year-old participants with a mean follow-up of 10.5 years (N = 14,569 of the Atherosclerosis Risk in Communities study), 11.6% had CVD and 5.0% had non-CVD death as first events, whereas among 65–84-year-old participants with 8.5 years of follow-up (N = 4237 of the Cardiovascular Health Study), these figures were 43.2% and 15.7%, respectively. Middle-aged blacks were significantly more likely than whites to experience any CVD as a first event; in older adults, this disparity disappeared, particularly after adjustment for CVD risk factors. However, there were no specific disparities among risk factors predicting individual outcomes.

The performance of Framingham CVD predictions in the Rotterdam Study, a prospective cohort study of individuals aged 55 years and older (N = 6004), was investigated by van Kempen et al. in 2014 [14]. The purpose was to validate the Framingham predictions of CVD, defined as the first occurrence of myocardial infarction, CHD death, or stroke during 15 years of follow-up, taking into account competing risks and just disentangling CVD into CHD and stroke. They calculated the cumulative incidence of CVD per individual by summation of the cause-specific hazard multiplied by the survival of the CVD event and the competing non-CVD death event at each failure time, a procedure that is much different from the Fine-Gray method, and concluded that Framingham CVD risk predictions perform well in the low- to intermediate-risk categories in the Rotterdam Study. Although disentangling CVD into CHD and stroke separately provided additional information about the contribution of CHD and stroke to total individual CVD risk, the follow-up was short, and no specific risk factors were associated individually with the 2 CVD components analyzed.

In 2016, we compared standard Cox and Fine-Gray models among 1677 middle-aged men in an Italian population study of CVD (part of the larger SCS) that reached 50 years of follow-up with the quasi-extinction of the population [7]. The incidence of either fatal or non-fatal cases in 50 years was used as a primary event, while deaths from any other cause, mutually exclusive from the primary events, were considered secondary events, and 10 selected risk factors were investigated. Cholesterol was significantly and positively related to the incidence of CHD compared with deaths from any other cause. Instead, when the primary events were deaths from any other cause and the competing events were CHD, cholesterol was inversely and age positively related. Other risk factors were predictors, including age, cigarettes, arm circumference (protective), systolic blood pressure, vital capacity (protective), corneal arcus, and diabetes, all documented by the Cox model (which also showed the role of cholesterol): which had common roles for both end-points. Later, CHD death versus 11 other causes of death, always comparing couples in contrast, were studied using the cumulative incidence function and the competing risk procedures to disentangle the differential role of risk factors for different end-points [8]. This enabled us to dissect, at least in part, the respective role that baseline covariates may have in segregating the probabilities of two types of death in contrast to each other. On that occasion, we advanced that mean serum cholesterol level was a specific risk factor for CHD deaths and clearly showed by applying the Fine-Gray model, at direct or inverse use (like conducted here), that comparing different end-points heavily influences the risk factor predictive capacity (among systolic blood pressure, cigarette smoking, and age).

In 2021, Khan et al. pooled and harmonized individual-level data from nine population-based cohorts in the United States [15]. All participants were free of clinical CVD at baseline, with available data on current smoking status, covariates, and CVD outcomes. They examined the association between smoking status and total CVD and CVD subtypes, including fatal and nonfatal CHD, stroke, CHF, and other CVD deaths. The study included 1,949,658 person-years of follow-up (observed from 1948 to 2017) during a median (interquartile range) of follow-up of 23 years (15–32 years), including 50.4% (among 106,165 adults) of women. In middle-aged men who reported smoking compared with those who did not smoke, competing hazard ratios (HRs) were higher for the first presentation being a fatal CVD event (HR, 1.79 [95% CI, 1.68–1.92]), with a similar pattern among women (HR, 1.82 [95% CI, 1.68–1.98]). Smoking was associated with earlier CVD onset by 5.1 and 3.8 years in men and women. Similar to the study of Feinstein et al. [13], a single risk factor was assessed [15], yet in men and women, the potential differential capacities of other risk factors remained unexplored.

A very good question was posed in the most recent original investigation on competing risks by Cooper et al. in 2022 [16]. In older adults, it was evaluated whether accounting for the competing risk of non-CVD death might improve the performance of CVD risk-prediction equations. All New Zealanders aged ≥65 years without a prior CVD hospitalization were enrolled. Using standard Cox and Fine-Gray approaches, sex-specific equations estimating the 5-year risk of a fatal or non-fatal CVD event were constructed. Among 360,443 people aged ≥65 years with 1,615,412 person-years of follow-up, 14.6% of men and 12.1% of women had a first CVD event, whereas 8.5% of men and 7.6% of women died from a non-CVD cause. Standard Cox models overestimated the 5-year CVD risk by around 1% overall and by 5–6% in the highest risk deciles. The mean predicted CVD risk from the Fine-Gray models approximated the observed risk overall, although slight underestimation occurred in some subgroups. This investigation insisted on calibration, which was better carried out by Fine-Gray than by Cox methods, and the specificities of different risk factor capabilities to predict outcomes were not investigated.

The SCS was the study identifying the Mediterranean diet [1,2,3], whose merits were clearly shown later when these eating habits were protective of 50-year follow-up CHD [23] and all-cause [24] mortalities in the 16 cohorts of the study. The approach was also refined and expanded by other contributions [25,26,27,28]. However, within the present analysis, the Mediterranean Diet does not have a direct function except to say that cohorts with lower long-term CHD mortality were those characterized by healthier dietary habits.

Finally, among the different biases and limitations that the present investigation may have, including the immortality bias alluded to above and the lack of consideration of the Mediterranean diet components among the covariates investigated, we should enumerate that women were not included based on the idea, at the time of enrollment, that too large cohorts should have been considered, a prohibitive shortcoming from the financial point of view and organization needs.

## 5. Conclusions

This life-long analysis of CVD mortality in extinct cohorts originally composed of middle-aged men in different countries and cultures establishes important aspects: (1) CHD mortality is bound by different risk factor relationships when put in competition with all other causes of death and with STHD. Serum cholesterol has crucial importance since, in both comparisons, its coefficients are positive and significant for CHD and negative and significant for the opposite end-points (all other causes of death and STHD). This highly points to an etiologic difference, inasmuch as dummy variables identifying the countries of SCS implicates that the low-cholesterol areas have negative coefficients, significant for CHD, while they become positive and significant in the other end-points; (2) Competing risk analyses (by Fine-Gray methods) are needed to assess risk factor capabilities in specific outcome types of CVD since they are different depending on outcome types considered (CHD versus stroke plus HDUE); (3) there are important differences among predictive covariates, in terms of presence/absence and opposition of the coefficients’ algebraic sign when the inversion is performed of primary versus the secondary outcome in case of Fine-Gray, what is much less evident or absent by Cox (Figure 1). However, the intimate mechanisms involved in these striking differences are still obscure; (4) findings from this analysis confirm those reported in a previous paper produced using the same material, using multiple but simpler approaches where the role of some risk factors—serum cholesterol in particular—explained different rates and risks for major subgroups of CVD mortality [4]. Moreover, they extend and confirm previous initial results on smaller cohorts [7,8]. Finally, the changing algebraic sign of country coefficients comparing direct versus inverse models might even suggest a possible “ecologic” [23,24] interpretation of the Fine-Gray analysis; (5) there is a clear-cut indication not to sum up or equate individual outcomes of CVD, which should be considered in all future investigations. 

## Figures and Tables

**Table 1:** Baseline (1958–61, 9063 men aged 40–59 years) risk factors expressed as mean and standard deviation (continuous variables) or proportion and standard error (discrete variables). See Table A1 in Appendix A for continuous risk factors expressed as medians and interquartile percentiles (25–75).

	USA	FINLAND	THE NETHERLANDS	ITALY	GREECE	JAPAN
Age (years)	49.4 (5.8)	49.4 (5.5)	49.9 (5.5)	49.1 (5.1)	49.3 (5.6)	49.8 (5.7)
Sedentary physical activity (PA), %	66.0 (0.9)	10.2 (0.7)	24.1 (1.4)	9.7 (0.7)	17.7 (1.1)	5.7 (0.7)
Moderate PA, %	34.0 (0.9)	15.7 (0.9)	64.6 (1.6)	22.1 (1.0)	33.7 (1.4)	29.4 (1.4)
Vigorous PA, %	0	74.1 (1.1)	11.3 (1.1)	68.2 (1.1)	48.6 (1.4)	64.9 (1.5)
Never smoker %	19.9 (0.8)	18.8 (1.0)	7.3 (0.8)	25.4 (1.1)	24.1 (1.2)	15.2 (1.1)
Ex-smoker %	21.1 (0.8)	18.3 (1.0)	18.2 (1.3)	13.6 (0.8)	15.9 (1.1)	9.9 (0.9)
Smoker (current), %	59.0 (1.0)	62.6 (1.2)	74.5 (1.5)	60.7 (1.2)	60.0 (1.4)	72.6 (0.9)
Body mass index (kg/m^2^)	25.2 (3.2)	23.7 (3.2)	24.0 (2.7)	25.2 (3.7)	23.1 (3.2)	22.0 (2.4)
Systolic Blood Pressure (mmHg)	139.2 (20.8)	143.9 (20.7)	144.4 (19.8)	143.6 (21.0)	136.2 (20.5)	135.0 (25.0)
Heart rate (beats/min)	72.1 (12.4)	67.7 (13.0)	72.6 (12.6)	71.3 (12.9)	64.6 (12.6)	63.0 (10.9)
Serum cholesterol mg/dL	240.3 (45.3)	261.0 (52.0)	235.5 (44.4)	201.7 (40.8)	205.4 (42.8)	165.2 (33.0)
CVD prevalence, %	11.3 (0.6)	19.4 (1.0)	7.7 (0.9)	5.4 (0.5)	4.9 (0.6)	0.6 (0.2)
ECG abnormalities, %	0.2 (0.03)	1.2 (0.3)	3.3 (0.6)	1.5 (0.3)	1.3 (0.3)	1.9 (0.4)

ANOVA across countries for continuous variables: *p* for Age = 0.0962; *p* for all other variables <0.0001. Chi-squared across countries for discrete variables: *p* for ECG = 0.024; *p* for all other variables <0.0001. See Methods [Risk factors: point (i)] for the definition of ECG abnormalities.

**Table 2:** Major CVD-type death rates (underlying cause per 1000) in 60 years in the six countries and their pool. All but a few men are deceased, so the rates in each column may be interpreted as 10 times the percentage of men dying of the given cause.

		CHD	Stroke	HDUE	STHD	Ratio CHD/STHD	CVD	ALL CAUSES
	N	Rates per 1000					Rates per 1000	
USA	2571	282	86	108	194	1.45	476	993
FINLAND	1677	352	101	61	162	2.18	514	998
The NETHERLANDS	878	267	84	65	149	1.79	416	998
ITALY	1712	164	132	120	252	0.65	416	998
GREECE	1215	136	164	137	300	0.45	436	998
JAPAN	1010	70	177	51	229	0.31	299	957
POOL	9063	228	118	95	213	1.07	441	992

Note that the 71 subjects censored are kept in the denominator. CHD = coronary heart disease; HDUE = Heart Diseases of Uncertain Etiology; STHD = Stroke + HDUE; CVD = CHD + Stroke + HDUE; N = men at baseline examination.

**Table 3:** Complete proportional hazards regression model with CHD as primary events and all other causes of death treated as competing risks (direct model) and the opposite with all other causes of death as primary events and CHD as competing risks (inverse model): *Approach 1* by Fine-Gray.

		A: Complete Direct Model				B: Complete Inverse Model				Test of Coefficients
Variable	Delta	Coeff	SE	HR	95% C.L	Coeff	SE	HR	95% C.L	
Age	5	0.0036	0.0042	1.02	0.98	0.0359	0.0023	**1.20**	**1.17**	**8.25**
					1.06				**1.22**	
Vigorous PA	reference	----	----	----	----	----	----	----	----	----
Moderate PA	1	0.0700	0.0677	1.07	0.94	0.0216	0.0323	1.02	0.96	1.22
					1.22				1.09	
Sedentary PA	1	0.1238	0.0757	1.13	0.98	0.0028	0.0033	1.00	0.92	1.45
					1.31				1.09	
Never smoker	reference	----	----	----	----	----	----	----	----	----
Ex-smoker	1	−0.0156	0.0734	0.98	0.85	0.0798	0.0382	**1.08**	**1.00**	0.78
					1.14				**1.17**	
Smoker	1	0.1223	0.0589	**1.13**	**1.01**	0.1182	0.0301	**1.13**	**1.06**	**3.64**
					**1.27**				**1.19**	
Body mass index	3.5	0.0170	0.0070	**1.06**	**1.01**	−0.0158	0.0045	**0.95**	**0.92**	0.15
					**1.11**				**0.98**	
Systolic blood pressure	20	0.0060	0.0012	**1.13**	**1.08**	0.0004	0.0007	1.01	0.98	**4.68**
					**1.18**				1.04	
Heart rate	13	−0.0018	0.0019	0.98	0.93	0.0035	0.0011	**1.05**	**1.02**	0.78
					1.02				**1.08**	
Serum cholesterol	50	0.0041	0.0005	**1.23**	**1.17**	−0.0022	0.0003	**0.90**	**0.87**	**3.34**
					**1.29**				**0.92**	
ECG abnormality	1	0.2219	0.1535	1.25	0.92	−0.0195	0.1072	0.98	0.79	1.08
					1.69				1.21	
Cardiovascular disease	1	0.3577	0.0700	**1.43**	**1.25**	−0.1629	0.0557	**0.85**	**0.76**	**2.18**
					**1.64**				**0.95**	
USA	1	−0.2301	0.0773	**0.79**	**0.68**	0.0538	0.0522	1.06	0.95	−1.89
					**0.92**				1.17	
FINLAND	reference	----	----	----	----	----	----	----	----	----
The NETHERLANDS	1	−0.2620	0.0904	**0.77**	**0.64**	0.0513	0.0585	1.05	0.94	−1.96
					**0.92**				1.18	
ITALY	1	−0.6341	0.0811	**0.53**	**0.45**	0.3251	0.0484	**1.38**	**1.26**	**−3.27**
					**0.62**				**1.52**	
GREECE	1	−0.8326	0.0928	**0.43**	**0.36**	0.2929	0.0477	**1.34**	**1.22**	**−5.17**
					**0.52**				**1.47**	
JAPAN	1	−1.2465	0.1363	**0.29**	**0.22**	0.4927	0.0551	**1.64**	**1.47**	**−5.13**
					**0.38**				**1.92**	

Units of measurement as in Table 1. Delta for computation of hazards rates of continuous variables roughly corresponding to 1 standard deviation of variables. In bold, significance is highlighted.

**Table 4:** Partial (excluding non-CVD deaths) proportional hazards regression model with CHD as primary events and STHD (other causes excluded) treated as competing risk (direct model) and STHD (other causes excluded) as primary events and CHD as competing risk (inverse model): *Approach 2* by Fine-Gray.

		A: Partial Direct Model				B: Partial Inverse Model				Test of Coefficients
Variable	Delta	Coeff	SE	HR	95% C.L	Coeff	SE	HR	95% C.L	
Age	5	−0.0072	0.0027	**0.96**	**0.94**	0.0080	0.0030	**1.04**	**1.01**	**−3.78**
					**0.99**				**1.07**	
Vigorous PA	reference	----	----	----	----	----	----	----	----	----
Moderate PA	1	−0.0020	0.0454	1.00	0.91	0.0056	0.0385	1.01	0.93	−0.13
					1.09				1.08	
Sedentary PA	1	0.0391	0.0508	1.04	0.93	−0.0359	0.0510	0.96	0.87	1.04
					1.15				1.07	
Never smoker	reference	----	----	----	----	----	----	----	----	----
Ex-smoker	1	−0.0099	0.0506	0.99	0.90	0.0258	0.0476	1.03	0.93	−0.51
					1.09				1.13	
Smoker	1	0.1085	0.0410	**1.11**	**1.03**	−0.1092	0.0379	**0.90**	**0.83**	**3.39**
					**1.21**				**0.97**	
Body mass index	3.5	0.0015	0.0048	1.01	0.97	−0.0001	0.0051	1.00	0.97	0.23
					1.04				1.04	
Systolic blood pressure	20	−0.0001	0.0007	1.00	0.97	0.0001	0.0007	1.00	0.97	−0.23
					1.03				1.03	
Heart rate	13	−0.0001	0.0012	1.00	0.97	0.0002	0.0013	1.00	0.97	−0.20
					1.03				1.04	
Serum cholesterol	50	0.0014	0.0003	**1.07**	**1.04**	−0.0019	0.0004	**0.91**	**0.87**	**6.80**
					**1.11**				**0.94**	
ECG abnormality	1	0.0289	0.0952	1.03	0.85	−0.0391	0.1030	0.96	0.87	0.48
					1.24				1.18	
Cardiovascular disease	1	0.0863	0.0396	**1.09**	**1.01**	−0.1326	0.0638	**0.88**	**0.77**	**2.92**
					**1.18**				**0.99**	
USA	1	−0.1247	0.0489	**0.88**	**0.80**	0.2196	0.0701	**1.25**	**1.09**	**−4.03**
					**0.97**				**1.43**	
FINLAND	reference	----	----	----	----	----	----	----	----	----
The NETHERLANDS	1	−0.0311	0.0544	0.97	0.87	0.0783	0.0896	1.08	0.91	−1.04
					1.08				1.29	
ITALY	1	−0.4491	0.0566	**0.64**	**0.57**	0.5178	0.0645	**1.68**	**1.48**	**−11.27**
					**0.71**				**1.90**	
GREECE	1	−0.6844	0.0729	**0.50**	**0.44**	0.6467	0.0640	**1.91**	**1.68**	**−13.72**
					**0.58**				**2.16**	
JAPAN	1	−0.9020	0.1118	**0.41**	**0.33**	0.6721	0.0720	**1.96**	**1.70**	**−11.84**
					**0.51**				**2.25**	

Units of measurement as in Table 1. Delta for computation of hazards rates of continuous variables roughly corresponding to 1 standard deviation of variables. In bold, significance is highlighted.

**Table 5:** Cox complete and partial approaches of CHD versus all other causes of death and CHD versus STHD (and the reverse models) and the *t*-tests to compare with the Fine-Gray results given in Table 3 and Table 4.

	Cox Direct Models				Cox Inverse Models				Test of Coefficients	
	CHD vs. OTHERS (Complete vs. Table 3A)		CHD vs. STHD (Partial vs. Table 4A)		OTHERS vs. CHD (Complete vs. Table 3B)		STHD vs. CHD (Partial vs. Table 4B)		Versus Those of Table 3	Versus Those of Table 4
Variable	Coeff ± SE	*t*-Test	Coeff ± SE	*t*-Test	Coeff ± SE	*t*-Test	Coeff ± SE	*t*-Test	A B	A B
Age	0.0678 ± 0.0044	**15.29**	0.0597 ± 0.0044	**13.48**	0.0986 ± 0.0025	**39.80**	0.1188 ± 0.0048	**24.54**	**10.51** **18.54**	**12.86** **19.48**
Vigorous PA	reference	-----	reference	-----	reference	-----	reference	-----	----- -----	----- -----
Moderate PA	0.1430 ± 0.0660	**2.17**	0.1212 ± 0.0673	1.80	0.4142 ± 0.0329	1.26	0.1579 ± 0.0619	**2.55**	0.77 0.43	1.52 **2.09**
Sedentary PA	0.1928 ± 0.0768	**2.51**	0.1689 ± 0.0778	**2.17**	0.0638 ± 0.0417	1.53	0.0890 ± 0.0778	1.14	0.64 1.46	1.40 1.34
Never smoker	reference	-----	reference	-----	reference	-----	reference	-----	----- -----	----- -----
Ex-smoker	0.0678 ± 0.0736	0.922	0.1069 ± 0.0738	1.45	0.1257 ± 0.0400	**3.14**	0.1410 ± 0.0718	**1.96**	0.80 0.83	1.30 1.34
Smoker	0.4259 ± 0.0595	**7.16**	0.5027 ± 0.0593	**8.48**	0.4198 ± 0.0317	**13.25**	0.3745 ± 0.0573	**6.54**	**3.63** **6.90**	**5.47** **7.04**
Body mass index	0.0094 ± 0.0073	1.28	−0.0050 ± 0.0073	−0.69	−0.0136 ± 0.004	**−3.21**	−0.0108 ± 0.0077	−1.41	−0.76 0.36	−0.75 −1.16
Systolic blood pressure	0.0121 ± 0.0011	**10.77**	0.0087 ± 0.0011	**7.76**	0.0071 ± 0.0007	**10.84**	0.0112 ± 0.0011	**9.77**	**3.77** **7.00**	**6.60** **8.18**
Heart rate	0.0018 ± 0.0018	0.97	0.0040 ± 0.0018	**2.21**	0.0052 ± 0.0010	**5.11**	0.0062 ± 0.0019	**3.23**	1.36 1.15	1.91 **2.56**
Serum cholesterol	0.0046 ± 0.0005	**9.78**	0.0035 ± 0.0005	**7.48**	−0.0001 ± 0.000	−0.44	0.0004 ± 0.0006	0.74	0.70 **4.87**	**3.75** **3.44**
ECG abnormality	0.3587 ± 0.1518	**2.36**	0.1105 ± 0.1522	0.73	0.2073 ± 0.0939	**2.21**	0.2142 ± 0.1589	1.35	0.63 1.59	0.45 1.34
Cardiovascular disease	0.5533 ± 0.0660	**8.39**	0.4983 ± 0.0658	**7.57**	0.2409 ± 0.0025	**5.16**	0.3206 ± 0.0842	**3.81**	**2.03** **5.55**	**5.37** **4.29**
USA	−0.3575 ± 0.0781	**−4.58**	−0.2720 ± 0.0795	**−3.42**	−0.1104 ± 0.049	**−5.16**	0.0336 ± 0.0944	0.36	−1.16 **−2.29**	−1.58 −1.58
FINLAND	reference	-----	reference	-----	reference	-----	reference	----	----- -----	----- -----
The NETHERLANDS	−0.4223 ± 0.0898	**−4.70**	−0.1853 ± 0.0919	**−2.02**	−0.1124 ± 0.055	**−2.03**	−0.1109 ± 0.1171	−0.95	−1.26 **−2.03**	−1.44 −1.28
ITALY	−0.5893 ± 0.0814	**−7.24**	−0.5250 ± 0.0816	**−6.44**	0.0809 ± 0.0457	1.77	0.4212 ± 0.0880	**4.78**	0.39 **−3.67**	−0.76 −0.89
GREECE	−1.0044 ± 0.0945	**−10.63**	−1.0339 ± 0.0962	**−10.74**	−0.1222 ± 0.047	**−2.58**	0.1796 ± 0.0896	**2.00**	−1.30 **−6.18**	**−2.89** **−4.24**
JAPAN	−1.1885 ± 0.1357	**−8.76**	−0.7740 ± 0.1368	**−5.66**	0.1587 ± 0.0539	**2.95**	0.8505 ± 0.1079	**7.88**	0.30 **−4.33**	0.72 1.38

Units of measurement as in Table 1. In bold, significance is highlighted.

**Figure A1:**
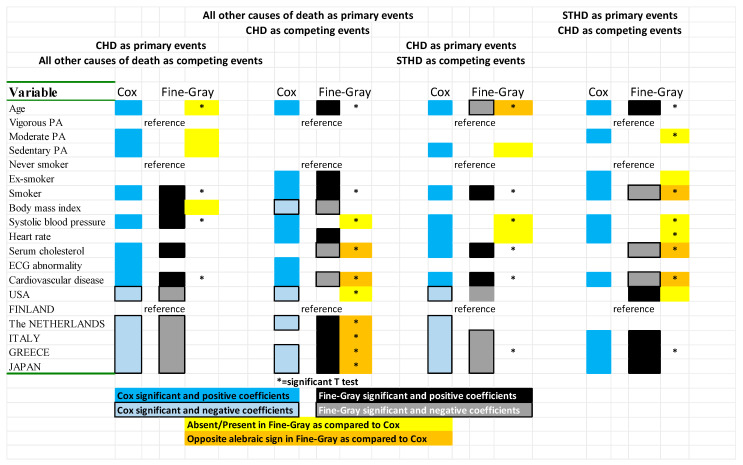
Synoptic graphical illustration of the differences between Cox and Fine-Gray models. This illustration in Appendix A is intended for color blindness; accordingly, red became blue and green became black with relevant brighter colors. Yellow and orange are identical to the original figure.

## Data Availability

The original data are not publicly available. However, research projects are evaluated centrally by an ad hoc committee.

## Appendix A

**Table A1:** Baseline continuous risk factors are expressed as medians and interquartile percentiles (25–75).

	USA	FINLAND	THE NETHERLANDS	ITALY	GREECE	JAPAN
Age	49	49	50	49	50	50
years	(44–55)	(45–54)	(45–54)	(45–53)	(45–54)	(45–55)
Body mass index	25.5	23.3	24.1	24.7	22.7	21.8
kg/m squared	(23.3–27.5)	(21.5–25.6)	(22.2–25.7)	(22.6–27.4)	(20.8–24.8)	(20.4–23.3)
Systolic blood pressure	135	140	140.5	140	132	130
mm Hg	(125–150)	(130–154)	(130–155)	(130–155)	(122–147)	(118–146)
Heart rate	71	65	71	70	63	62
beats/min	(63–80)	(58–75)	(64–80)	(62–78)	(56–70)	(55–69)
Serum cholesterol	236.3	258	232	198	200	164
mg/dL	(210–268)	(225.5–291)	(206–227.3)	(175–224)	(177–229)	(143–184)

Kruskal-Wallis ANOVA for medians: *p* of chi squared = 0.0596 for age; <0.0001 for other variables.
